# Abnormal eye movements in parkinsonism: a historical view

**DOI:** 10.1590/0004-282X-ANP-2020-0406

**Published:** 2021-05-01

**Authors:** Tereza Ko, Augusto Mädke Brenner, Nicholas Pili Monteiro, Mariana Severo Debastiani, Alberto Chitolina Nesello, Arlete Hilbig

**Affiliations:** 1 Universidade Federal de Ciências da Saúde de Porto Alegre Faculdade de Medicina Porto Alegre RS Brazil Universidade Federal de Ciências da Saúde de Porto Alegre, Faculdade de Medicina, Porto Alegre RS, Brazil.; 2 Universidade Federal de Ciências da Saúde de Porto Alegre Departamento de Clínica Médica Porto Alegre RS Brazil Universidade Federal de Ciências da Saúde de Porto Alegre, Departamento de Clínica Médica, Porto Alegre RS, Brazil.; 3 Irmandade Santa Casa da Misericórdia de Porto Alegre Porto Alegre RS Brazil Irmandade Santa Casa da Misericórdia de Porto Alegre, Serviço de Neurologia, Porto Alegre RS, Brazil.

**Keywords:** Eye Movements, History of Medicine, Parkinson Disease, Parkinsonian Disorders, Movimentos Oculares, História da Medicina, Doença de Parkinson, Transtornos Parkinsonianos

## Abstract

Parkinson's disease (PD), known since ancient times as *paralysis agitans*, was studied and described by James Parkinson in 1817 in his work “An Essay on the Shaking Palsy”. Fifty years later, Charcot and his students delved into the disease, naming it as we know it today, as well as defining the classic disease and its variants. One of these students, Arthur Dutil, addressed patients’ abnormal eye movements. Nowadays, it is known that the differential diagnosis of PD is relevant for prognosis, treatment and research, and, despite the advances in the area, it remains largely clinical. The relevance of the eye movement exam has grown along with the history of PD and it has proved to be an excellent tool for the differential diagnosis of parkinsonism. Additionally, it can become a support to identify different types of genetic PD and be useful for improving early recognition of cognitive decline in patients with PD.

## RESUMO

A doença de Parkinson (DP), conhecida desde a antiguidade como *paralysis agitans*, foi estudada e descrita por James Parkinson em 1817 em seu trabalho *An Essay on the Shaking Palsy*. Cinquenta anos depois, Charcot e seus alunos aprofundaram os estudos na doença, nomeando-a como a conhecemos atualmente, bem como definindo a doença clássica e suas variantes. Um desses estudantes, Arthur Dutil, investigou os movimentos oculares anormais nos pacientes. Hoje, sabe-se que o diagnóstico diferencial da DP é relevante para o prognóstico, tratamento e pesquisa, e, apesar dos grandes avanços na área, ainda permanece amplamente clínico. A relevância do exame de movimentação ocular cresceu com a história da DP e este se mostrou uma excelente ferramenta para o diagnóstico diferencial do parkinsonismo. Além disso, pode se tornar um auxílio para identificar diferentes formas de DP genética e útil para melhorar o reconhecimento precoce do declínio cognitivo em pacientes com DP.

In the year of 1817, James Parkinson (1775–1824), a physician and geologist who lived near London, England, published his monograph “An Essay on the Shaking Palsy”^1^. Within his work, he described greatly what had been seen a long time before him by individuals like Galen^2^^,^^3^, known at that time as *paralysis agitans*. Approximately fifty years after his publication, Jean-Martin Charcot (1825–1893) and his students at the Salpêtrière Hospital in Paris, France, complemented the analyses of the disease by naming it as we know today, Parkinson's disease (PD), and distinguishing it from other similar diseases at that time, as multiple sclerosis^2^^,^^4^. Furthermore, Charcot and his students identified atypical variants of what was defined as the classic PD. Those variants were called PD without tremor, PD with hemiplegia, and PD with extended posture^2^^,^^5^. Nowadays, these variants are known to be atypical parkinsonism, as progressive supranuclear palsy (PSP), multiple system atrophy (MSA) and corticobasal degeneration (CBD)^2^.

Among different predominant features of PD seen at that time, as bradykinesia and rigidity, one of Charcot's students, Arthur Dutil, described, in 1889, a woman with parkinsonian features and abnormal eye movements. Although no clear description of supranuclear palsy was reported, the presence of retrocollis and erect trunk suggest a PSP phenotype^6^. Prior to Dutil, in 1838, an interesting report was made by Marshall Hall, when he wrote “a peculiar lateral rocking motion of the eyes” regarding a 28-year-old male thought to be a case of PD^7^.

Throughout the first half of the 20^th^ century, authors, including E. Krebs, in 1925, and A. Bielschowsky, in 1935, have described abnormal eye movements in patients with PD^8^. Fisher, in 1924, and Portman, in 1932, found first degree spontaneous nystagmus that appears when the patient gazes in the direction of the fast component to be frequent in PD^9^^,^^10^. After the influenza epidemic of 1916–1917, there was an important increase in cases of postencephalitic parkinsonism^2^. Therefore, many studies were based on the observation of postencephalitic parkinsonism cases^11^, which occur with characteristic oculogyric crisis, pupillary changes, and gaze palsies^12^. Alexandre, in 1939, and Fracasso and Palatini, in 1941, however, found first degree spontaneous nystagmus to be rare in patients with PD.

**Figure 1:**
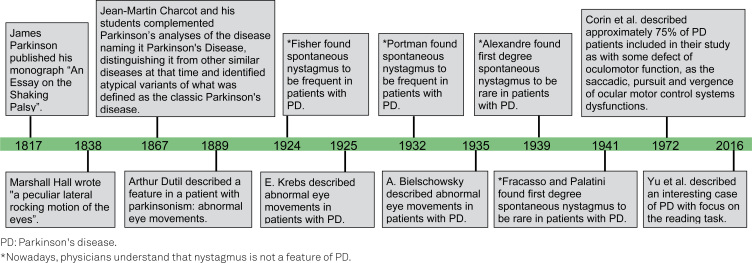
Parkinson's disease and Parkinsonisms’ timeline.

Research on abnormal eye movements in parkinsonian disorders was intensified in the second half of the 20^th^ century (Figure 1). In 1972, Corin et al.^8^ published an important case-control study on oculomotor dysfunction in PD^8^ and described some dysfunctions as the saccadic, pursuit, and vergence in approximately 75% of patients. As the criteria for PD was not mentioned in these ancient studies, we can assume that at least part of their sample had parkinsonian syndrome other than PD as we define today.

By the year of 2020, much has been known about the association of parkinsonism and abnormal eye movements, their role in differential diagnosis of parkinsonian disorders, and their potential aid to better elucidate diseases pathophysiology.

Among parkinsonism and movement disorders, PD appears to have less prominent oculomotor abnormality^13^ (Table 1). Nevertheless, hypometric voluntary saccades, saccadic intrusions during eye fixation, impaired vertical soft search/tracking movements and convergence insufficiency can be seen in PD and worsen as disease progresses^13^^,^^14^. In comparison, PSP patients present oculomotor disorders in early disease stage, including frequently slow vertical saccades with normal speed horizontal saccades (although often hypometric) that also become slow with disease progression, and impaired convergent ocular movements and linear vestibulo-ocular reflex. PSP is liable to be confused with CBD in consequence of the vertical paralysis of supranuclear gaze^13^^,^^14^. However, it was observed that CBD presents with significant increased latency of horizontal saccades, while PSP presents with a decrease of saccade speed^13^^,^^14^.

**Table 1:** Ocular motor abnormalities in parkinsonism, adapted from Jung^13^.

Diagnosis	Parkinson's disease	Progressive supranuclear palsy syndrome	Corticobasal syndrome	Multiple systemic atrophy
Saccadic intrusions	+	++	+	+
Vertical saccades	Hypometric	Slowed, early	Impaired, late	Hypometric
Horizontal saccades	Hypometric	Slowed, late	Delayed	Hypometric
Blepharospasm or eyelid apraxia	Very rare	Common	Common	Rare
Smooth pursuit	Mild	Severe	Mild	Moderate

In 2016, Yu et al.^15^drew attention to reading disturbance in PD analyzing a 40-year-old man with a six-year history of disease. The man was on dopamine-replacement therapy, had high schooling levels and good eye movement control. His ability in making alternating fixations and saccades during number reading was normal on the neuro-ophthalmic examination. Nevertheless, his word reading was significantly slowed and abnormal due to increased number of saccades, smaller saccade amplitudes, increased number of regressive saccades and longer fixation durations.

Waldthaler et al.^16^, in 2018, also characterized eye movements during reading in PD patients. They suggest that eye movements during reading reflect cognitive impairment in PD. Wong et al.^17^ had explored the relationship between the eye movement parameters and performance in cognitive tests in multiple domains. As a result, it was found that the prolonged duration of fixation was associated with poorer performance in verbal fluency, visual and verbal memory. Gorges et al.^18^ analyzed eye movements using video-oculography and correlated the findings with functional brain mapping obtained from task-based/no-task-based functional magnetic resonance imaging (MRI). The results show that the worst oculomotor performance is associated with the severity of the functional impairment of the brain structures involved with the disease. These findings may allow further exploration of the use of eye movement parameters as a clinical proxy for cognitive function markers in patients with PD. Similarly, Barbosa et al. described an association between the saccadic direction error with impulse-compulsive behavior in PD patients^19^.

Another interesting line of study is the analysis of the different inherited parkinsonian syndromes and their dysfunction profiles in the basal ganglia. The study, carried out in 2017 by Pretegiani et al.^20^, compared the pattern of alteration of attributes of horizontal saccades, vertical saccades and antisaccades in PD and different forms of genetic parkinsonism, such as PARK1, PARK2, PARK6 and PARK9 inherited forms of PD, in addition to neurodegeneration with brain iron accumulation (NBI) and Gaucher Disease, among others. The results show that each one of the mentioned diseases may have a different pattern of changes in the saccadic movements. Such a study allows important inferences to be made about the different dysfunctions presented by the basal ganglia in these syndromes.

In conclusion, the differential diagnosis of PD is relevant for prognosis, treatment, and research, and, despite major advances in the field, it still remains largely clinical. The relevance of eye movement examination has grown alongside the history of PD and has proven to be an excellent tool for differential diagnosis of parkinsonism^13^^,^^21^. Moreover, it may become an aid to identify different forms of genetic PD, and useful to improve early recognition of cognitive decline in PD patients.
